# Wide Range Applications of Spirulina: From Earth to Space Missions

**DOI:** 10.3390/md20050299

**Published:** 2022-04-28

**Authors:** Giacomo Fais, Alessia Manca, Federico Bolognesi, Massimiliano Borselli, Alessandro Concas, Marco Busutti, Giovanni Broggi, Pierdanilo Sanna, Yandy Marx Castillo-Aleman, René Antonio Rivero-Jiménez, Antonio Alfonso Bencomo-Hernandez, Yendry Ventura-Carmenate, Michela Altea, Antonella Pantaleo, Gilberto Gabrielli, Federico Biglioli, Giacomo Cao, Giuseppe Giannaccare

**Affiliations:** 1 Interdepartmental Centre of Environmental Science and Engineering (CINSA), University of Cagliari, Via San Giorgio 12, 09124 Cagliari, Italy; faisgiacomo@gmail.com (G.F.); alessandro.concas@unica.it (A.C.); giacomo.cao@unica.it (G.C.); 2 Department of Biomedical Science, University of Sassari, Viale San Pietro 43/B, 07100 Sassari, Italy; alessia_manca@hotmail.it (A.M.); apantaleo@uniss.it (A.P.); 3 Unit of Maxillofacial Surgery, Head and Neck Department, ASST Santi Paolo e Carlo Hospital, University of Milan, Via Antonio di Rudinì 8, 20142 Milan, Italy; federico.bolognesi5@unibo.it (F.B.); federico.biglioli@unimi.it (F.B.); 4 Department of Biomedical and Neuromotor Sciences, University of Bologna, Via Zamboni 33, 40126 Bologna, Italy; 5 Department of Ophthalmology, University Magna Grecia of Catanzaro, Viale Europa, 88100 Catanzaro, Italy; mborselli93@gmail.com; 6 Department of Mechanical, Chemical and Materials Engineering, University of Cagliari, Via Marengo 2, 09123 Cagliari, Italy; 7 Nephrology, Dialysis and Transplant Unit, IRCCS-Azienda Ospedaliero Universitaria di Bologna, University of Bologna, Via Giuseppe Massarenti 9, 40138 Bologna, Italy; marco.busutti@aosp.bo.it; 8 Department of Neurosurgery, Fondazione IRCCS Istituto Neurologico Carlo Besta, University of Milan, Via Celoria 11, 20133 Milan, Italy; gbroggi@gmail.com; 9 Columbus Clinic Center, Via Michelangelo Buonarroti 48, 20145 Milan, Italy; 10 Abu Dhabi Stem Cells Center, Al Misaha Street, Rowdhat, Abu Dhabi, United Arab Emirates; pierdanilo.sanna@gmail.com (P.S.); yandy.castillo@adscc.ae (Y.M.C.-A.); rene.rivero@adscc.ae (R.A.R.-J.); antonio.bencomo@adscc.ae (A.A.B.-H.); yendry.ventura@adscc.ae (Y.V.-C.); 11 TOLO Green, Via San Damiano 2, 20122 Milan, Italy; m.altea@tologreen.it (M.A.); gil.gabrielli@gmail.com (G.G.); 12 Center for Advanced Studies, Research and Development in Sardinia (CRS4), Loc. Piscina Manna, Building 1, 09050 Pula, Italy

**Keywords:** Spirulina, healthcare, space missions, medicine applications, microgravity effects

## Abstract

*Spirulina* is the most studied cyanobacterium species for both pharmacological applications and the food industry. The aim of the present review is to summarize the potential benefits of the use of Spirulina for improving healthcare both in space and on Earth. Regarding the first field of application, Spirulina could represent a new technology for the sustainment of long-duration manned missions to planets beyond the Lower Earth Orbit (e.g., Mars); furthermore, it could help astronauts stay healthy while exposed to a variety of stress factors that can have negative consequences even after years. As far as the second field of application, Spirulina could have an active role in various aspects of medicine, such as metabolism, oncology, ophthalmology, central and peripheral nervous systems, and nephrology. The recent findings of the capacity of Spirulina to improve stem cells mobility and to increase immune response have opened new intriguing scenarios in oncological and infectious diseases, respectively.

## 1. Introduction

*Arthrospira platensis* and *Arthrospira maxima* (also known more generically as *Spirulina* for its spiral or helical shape), which belong to the Microcoleaceae family, are the most studied cyanobacterium species in pharmacological applications and the food industry [1,2,3,4]. Cyanobacteria are Gram-negative bacteria that have played an important role in the evolution of primitive Earth and the biosphere; they have been responsible for the oxygenation of the atmosphere and oceans since the Great Oxidation Event (GOE) around 3 billion years ago [5]. These microorganisms have been classified as blue-green algae for their color due to the production of the pigment phycocyanin. Cyanobacteria have been found in various ecological niches, from freshwater to the oceans, on soils, rocks, and in environments with extreme physicochemical characteristics [6]. *Spirulina* is an obligate photoautotrophic filamentous species and has a characteristic helical shape. This cyanobacterium has a prokaryotic organization with a multilayered cell wall, ribosomes, numerous inclusions, and a lamellar photosynthetic system. 

Cyanobacteria thrive naturally in alkaline waters, rich in minerals, under temperatures ranging from 35 to 40 °C. Its filaments can reach the size of 0.5 mm in length. The helical shape of the filament and the presence of gas-vacuoles inside the cells make it form floating mats. Several species of *Spirulina* are present in nature, but the most studied and used species are *Spirulina platensis* (*S. platensis*) and *Spirulina maxima* (*S. maxima*) [7]. Since the 70s, Spirulina has been considered a rich food source due to its high content of macro-and micronutrients. In fact, it is an excellent source of proteins, vitamins, fatty acids, minerals, photosynthetic pigments, and several secondary metabolites (Table 1) [8].

Depending on how it is grown, it can contain up to 60% of dry weight (dw) proteins consisting of essential amino acids such as leucine, isoleucine, valine, tryptophan, methionine, phenylalanine, lysine, and threonine [9,10,11,12]. The protein content is of high quality, with a biological value of 75% and a high digestibility (83–90%) because *Spirulina* cells display a fragile and easily digestible murein envelope instead of cellulose walls [13,14,15]. Approximately 60% of the total protein content consists of phycobilisomes, composed of C-phycocyanin (C-PC) and allophycocyanin (A-PC), relevant antioxidant, anti-inflammatory, antitumor, and immunostimulant compounds, as well as ingredients for the cosmetic, food, pharmaceutical, and nutraceutical industries [16,17,18]. About 7–16% of the dry weight consists of lipids [19,20]. These include monounsaturated fatty acids such as oleic acid, saturated fatty acids (SFA) such as the more abundant palmitic, arachidic, and myristic acids, and the omega-6 polyunsaturated fatty acids (ω-6 PUFAs) arachidonic (AA) and γ linolenic acid (GLA), a potent immunoprotective and precursor to prostaglandins and leukotrienes [19,21,22,23]. Fatty acids of Spirulina also include stearidonic acid (SDA), eicosapentaenoic acid (EPA), and docosahexaenoic acid (DHA) [24]. Spirulina contains different lipophilic pigments which have different bioactive properties. Among these, the most abundant are chlorophyll-a (9–12% of the lipid fractions) and β-carotene. Xanthophylls, echinenone, myxoxanthophyll, zeaxanthin, canthaxanthin, β-cryptoxanthin, and oscillaxanthina are other terpenoids present in Spirulina [25]. Regarding vitamins, Spirulina contains provitamin A (β-carotene), vitamin B1 (thiamine), B2 (riboflavin), B3 (nicotinamide), B6 (pyridoxine), B9 (folic acid), B12 (cyanocobalamin), vitamin C, vitamin D, and vitamin E (tocopherol). Spirulina is also a source of several minerals, including iron, zinc, potassium, copper, manganese, magnesium, phosphorus, and calcium. [8,19,23,26]. It has been demonstrated that, depending on how it is grown, Spirulina carbohydrates, including glucose, fructose, sucrose, glycerol, sorbitol, and mannitol, can increase from 15 to 20% dw [26]. In addition, myo-inositol, a carbohydrate source of organic phosphorus, nitrogen, calcium spirulan (Ca-SP), and the polysaccharide immunomodulator called Immulina, can be found in Spirulina biomass [8,27]. Moreover, Spirulina species can adapt to environmental changes and grow very rapidly depending on the availability of nutrients and climatic factors [28,29]. Through photosynthesis, these strains can fix N_2_ and CO_2_ while producing O_2_ and organic compounds such as polysaccharides, lipids, carotenoids, proteins, and vitamins, which play a key role in the food, cosmetics, and pharmaceutical industries [30].

**Figure 1:**
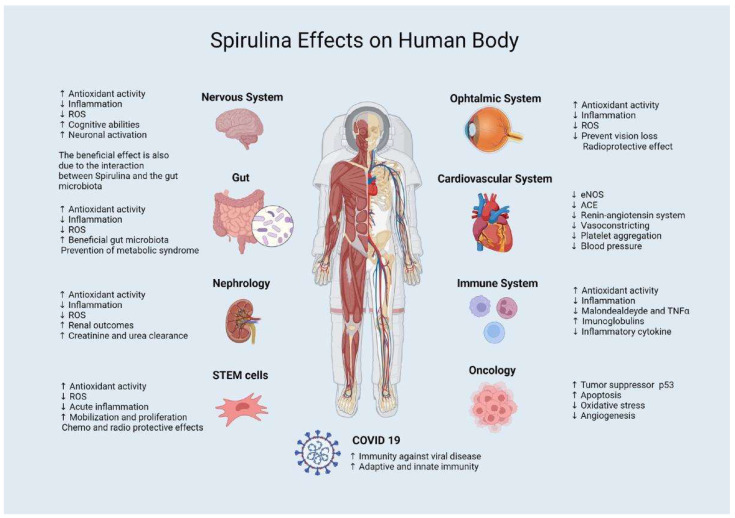
An overview of the effects of *Spirulina* on the various districts of the human body. Created with BioRender.com (accessed on 8 March 2022).

Spirulina has been known since ancient times as a superfood. Aztecs collected it from the alkaline Lake Texcoco, Mexico, as an integral part of their diet [31,32]. The safety of Spirulina has been demonstrated by many toxicological studies both in chronic or acute administration in the liver, kidneys, reproductive system, and human physiology [33,34,35,36,37]. Currently, it is listed by the US Food and Drug Administration (FDA) in the category “generally recognized as safe” (GRAS) [38]. Spirulina has been declared the world’s first superfood, with a complete nutritional profile [39] and the best “food of the future” for its eco-sustainability. Nowadays, Spirulina is produced industrially under controlled conditions [23]. In fact, the growth and productivity of this microorganism depend on several factors such as nutrient concentration, temperature, the light spectrum, intensity, and pH, which also influence its biochemical composition [40,41]. Furthermore, Spirulina is used in medicine to treat different health conditions thanks to bioactive compounds such as antioxidants, immuno-stimulants, anti-inflammatory, antibacterial, antiviral, antitumor, antiallergic, antidiabetic, including phenolics, phycobiliproteins, and chlorophyll [20,21,42,43,44]. These and other compounds from Spirulina are believed to prevent or treat a wide range of medical conditions [23,45] (Figure 1). 

For these reasons, space agencies such as the National Aeronautics and Space Administration (NASA) have promoted it as a food and supplement for astronauts during their space missions since small amounts are able to provide different nutrients and protective effects [46,47,48,49,50]. In fact, during their stay in space, astronauts are exposed to a variety of stress factors that can have negative health consequences [51,52].

The space environment harms different districts of the astronaut’s body due to several factors, such as micro-gravity and oxidative stress induced by radiation [53]. On Earth, the cells are constantly subjected to the action of Reactive Oxygen Species (ROS), normally produced as a result of cellular metabolism and exposure to environmental stresses such as X-rays, UV, pollution, xenobiotics, etc. The damage is directly proportional to the ROS produced. This production is mitigated by the activation of cellular detoxification mechanisms that physiologically protect cells. When ROS-dependent stress exceeds the body’s physiological defenses, oxidative damage occurs to cells. The extraterrestrial environment determines an increase of ROS, causing damage to cellular lipid membranes, mitochondria, proteins, and DNA [54]. Microgravity and radiation affect several molecular systems and mechanisms such as repair, replication, transcription, and protein expression [55]. 

In this context, Spirulina, with its antioxidant activity, is able to activate cellular antioxidant enzymes and inhibit lipid peroxidation and DNA damage, thus eliminating free radicals and increasing superoxide dismutase and catalase enzyme activity. Spirulina also possesses immunomodulatory and anti-inflammatory properties that may play a preventive role against potential astronauts’ pathologies [17].

In the same context, *Spirulina* has been designated to be placed in Environmental Control and Life Support Systems (ECLSS) in the Micro-Ecological Life Support System Alternative project (MELiSSA), which allows the production of water, oxygen, food, and health supplements by the total recycling of the space station crew’s metabolic waste, including the exhaled CO_2_ into the cabin [56,57,58,59,60,61].

To date, the effects of using Spirulina as an adjunct to classical therapies for the most common diseases on Earth are well known in the literature, while the effects of Spirulina on human health in space have not yet been studied [45,62]. In this review, we will discuss the effects of Spirulina on major diseases that can affect humans both on Earth and in space.

The aim of the present review is to summarize the potential benefits of the use of Spirulina, such as food or supplements, to improve the health of astronauts during space missions.

## 2. Clinical Applications on Earth

### 2.1. Probiotic Effect

Microbial modulation activities have been reported in vitro, suggesting that the combination of Spirulina and probiotics may represent a new strategy to enhance the growth of the beneficial gut microbiota. Spirulina can inhibit the growth of some Gram-negative (*Escherichia coli*, *Pseudomonas aeruginosa* and *Proteus vulgaris*) and Gram-positive (*Staphylococcus aureus*, *Bacillus subtilis* and *Bacillus pumilus*) bacteria [63]. In fact, *Spirulina* produces extracellular metabolites with antibacterial activity. On the contrary, low (minimum inhibitory concentrations, MIC ≥ 512 μg/mL) or no inhibitory effect was found against other bacteria (*Pseudomonas aeruginosa*, *Salmonella typhimurium*, and *Klebsiella pneumoniae*) [64]. Moreover, it has been reported that extracellular products of *Spirulina*, obtained from a culture in the late exponential stage and separated by filtration, significantly promote the in-vitro growth of lactic acid bacteria (*Lactococcus lactis*, *Streptococcus thermophilus*, *Lactobacillus casei*, *Lactobacillus acidophilus*, and *Lactobacillus bulgaricus*) [65]. It is largely established that drastic changes of microbiota composition occur in several gastrointestinal, immunological, and metabolic diseases [66,67]. In many microbiota-related diseases, including Inflammatory Bowel Disease (IBD), it is well known that a strong unbalanced ratio among the genera of potentially protective bacteria and normal anaerobic bacteria is present. In particular, *Bacteroides* sp., *Eubacterium* sp., and *Lactobacillus* sp. are significantly decreased [68]. Spirulina could represent an alternative strategy to symbiotic formulations [63]. The latter seems to be more effective than probiotics alone in the prevention of dysbiosis associated with immune-mediated, inflammatory, and dysmetabolic diseases. Research on the advanced medical applications of Spirulina and derived products in the treatment of infectious diseases caused by Gram-positive organisms are growing. Due to the health benefits promoted, it is gaining more and more interest, especially in of dietary supplements, where it is used as a powder or consumed as capsules or tablets [63]. This evidence suggests that Spirulina may be useful for improving animal and human health by changing the gut microbiota composition and promoting beneficial bacterial growth [68].

### 2.2. Antihypertensive Effect

Among the main causes of cardiovascular diseases, such as atherosclerosis, cardiac hypertrophy, heart failure, and hypertension, oxidative stress and inflammation are of primary importance. Overproduction of ROS because of oxidative stress has been observed in cardiovascular disease conditions [69]. In addition, evidence indicates that Low Density Lipoprotein (LDL) oxidation is essential for atherogenesis [70,71]. On the other hand, the microenvironment within the atherosclerotic lesion is proinflammatory. In addition to being a disorder of lipid metabolism, atherosclerosis is now recognized as a chronic inflammatory disease [72,73]. Accumulating evidence demonstrates that excessive inflammation within the arterial wall is a risk factor for cardiovascular diseases and can promote atherogenesis. Agents with antioxidant and/or anti-inflammatory activity may prove beneficial in combating cardiovascular diseases. Vascular aging is characterized by an increase in arterial stiffness and remodeling of the arterial wall with a loss of elastic properties. Silicon is an essential trace element highly present in arteries. The effects of nutritional supplementation with silicon-enriched Spirulina (SpSi) on arterial system structure and function in hypertension have been evaluated. Collagen and elastin levels were increased in association with extracellular matrix degradation decrease. The beneficial effects of SpSi supplementation evidenced here may be attributable to Spirulina enrichment and offer interesting opportunities to prevent cardiovascular risks [74]. Spirulina can be effective as a blood pressure-reducing agent by increasing endothelial nitric oxide synthase (eNOS), inhibiting angiotensin-converting enzyme (ACE), suppressing renin-angiotensin system, vasoconstricting metabolites, and platelet aggregation [74]. In the future, the use of Spirulina could have fundamental implications for hypotensive therapies in cardiology [63].

### 2.3. Antioxidant and Anti-Inflammatory Effects

Spirulina contains several active components, notably phycocyanin and β-carotene, with potent antioxidant and anti-inflammatory activities [64]. As anti-inflammatory activities, phycocyanin inhibits proinflammatory cytokine formation, such as TNFα, suppresses cyclooxygeanase-2 (COX-2) expression and decreases prostaglandin E(2) production [75]. Another component of Spirulina, β-carotene, has been reported to have antioxidant and anti-inflammatory activities. It was found that β-carotene is able to protect cells against singlet oxygen-mediated lipid peroxidation [76]. ROS also contribute to vascular dysfunction and remodeling through oxidative damages in endothelial cells [77]. In a recent in vitro study [78], the antioxidant and anti-inflammatory properties of four different Spirulina preparations were evaluated with a cell-free as well as a cell-based assay. It was found that Spirulina dose-dependently inactivated free superoxide radicals generated during an oxidative burst [78]. Supplements seem to affect innate immunity more effectively than acquired immunity, promoting the activity of natural killer cells. There are wide margins of use to exploit this capacity improves markers of oxidative stress and NK activity in healthy subjects and CD4+ counts in HIV+ patients. In the field of chemotherapy therapies, the effect of Spirulina against the hepatotoxicity of methotrexate has been studied. Administration of a high dose of Spirulina for 21 days prior to methotrexate treatment reduced malondialdehyde and tumor necrosis factor α [79]. In addition, the antioxidant activity of Spirulina has been associated with anti-inflammatory effects (see paragraph 3). Although the antioxidant effect of Spirulina is confirmed by some studies [17,79], the concerted modulation of antioxidant and inflammatory responses, suggested by in vitro and animal studies, requires greater confirmation in humans. Most of the studies on Spirulina are on animal samples [17,79]. From these studies, it was shown that Spirulina is an antioxidant and has a protective effect against damage to DNA. The immunostimulant effect is exerted by Spirulina in stimulating the production of immunoglobulins and the downregulation of inflammatory cytokine-producing genes [80]. Spirulina is generally considered safe for human consumption, supported by its long history of use as a food source and its favorable safety profile in animal studies. However, rare cases of side effects in humans have been reported [62].

### 2.4. Antiviral Effect

There are no in vivo studies providing strong evidence supporting the possible antiviral properties of Spirulina. However, Spirulina inhibits the in vitro replication of several enveloped viruses, including Herpes simplex type I, human cytomegalovirus, the measles and mumps virus, influenza A, and Human immunodeficiency virus-1 virus (HIV-1) [45,81]. L-asparaginase (L-AsnA) is an enzyme present in the composition of *Spirulina*. L-AsnA enzyme demonstrated good antiviral activity against the Coxsackie B3 (CSB3) virus in a dose-dependent manner. The antiviral mode of action is likely due to its capability of inhibiting attachment, blocking the adsorption and penetration event of the viral replication cycle with 89.24%, 72.78%, and 72.78%, respectively [82]. The ability of Spirulina-based nutraceuticals to boost immunity against viral diseases has already been reported clinically. Spirulina-based nutraceuticals boost adaptive and innate immunity, and bioactive compounds, such as ACE inhibitor peptides, phycobiliproteins, sulfated polysaccharides, and calcium spirulan, can serve as antiviral agents. The presence of these molecules indicates its potential role in resisting infection and COVID-19 disease progression (see Section 6) [83]. A recent study has shown that the hot water extract of Spirulina inhibited the infection of herpes simplex virus type 2 (HSV-2), pseudorabies virus (PRV), human cytomegalovirus (HCMV), and HSV-1. For adenovirus, the inhibition was less than 20%, and no inhibition was found for the measles virus, subacute sclerosing panencephalitis virus (SSPE), vesicular stomatitis virus (VSV), poliovirus 1, or rotavirus SA-11. The highest antiviral activity was for HSV-2. The antiviral activity was not due to a virucidal effect. Herpesvirus infection was inhibited at the initial events (adsorption and penetration) of the viral cycle [84].

### 2.5. Antihistamine Effect

Spirulina exhibits anti-inflammatory properties by inhibiting the release of histamine from mast cells. In a recent study [80], individuals with allergic rhinitis were fed daily, either with placebo or Spirulina, for 12 weeks. The study showed that a high dose of Spirulina significantly reduced IL-4 levels, demonstrating the protective effects on allergic rhinitis. Spirulina consumption significantly improved the symptoms and physical findings compared with placebo, including nasal discharge, sneezing, nasal congestion, and itching. Moreover, Spirulina increases the IgA levels on the surface of mucous membranes. Two peptides, LDAVNR (P1) and MMLDF (P2), purified from enzymatic hydrolysate of Spirulina, have been reported to be effective against allergic rhinitis. It was revealed that P1 and P2 exhibited significant inhibition of mast-cell degranulation via decreasing histamine release and intracellular Ca^2+^ elevation. The inhibitory activity of P1 was found due to the blockade of calcium- and microtubule-dependent signaling pathways. Meanwhile, the inhibition of P2 was involved in the suppression of phospholipase Cγ activation and reactive oxygen species production. These findings indicate that peptides P1 and P2 from Spirulina may be promising candidates for antiallergic therapeutics, contributing to the development of bioactive food ingredients to ameliorate allergic diseases [85].

## 3. Applications in Ophthalmology

### 3.1. Therapeutic Effect of Spirulina in Corneal Inflammation 

It has been established that Spirulina extract reduces alkaline burn-induced inflammation more effectively than amniotic membrane extract (AME). For this reason, it can be used for the therapy of corneal diseases involving neovascularization and inflammation. Other implications of the antioxidant effect of Spirulina can be found in the protection of the neuroepithelium. It should be highlighted that patients who assume Spirulina recorded a lower percentage of thinning of the layer of photoreceptors and death of such cells. In addition, the increase in retinal ROS levels after exposure to light has been reduced by Spirulina supplementation. Among the future implications of Spirulina should be considered the potential use in the form of a nutritional supplement to prevent vision loss related to oxidative damage [86,87].

### 3.2. Radioprotective Effect of Spirulina on Lacrimal Glands

The radioprotective effect of Spirulina in patients that needed radiation treatments (RAI) for other purposes has been studied. The radioprotective effect of Spirulina on lacrimal glands was evaluated with histopathological and cytopathological analysis. The evaluation was assessed before and after treatment with Spirulina, and it was found to decrease the level of oxidation after RAI [88].

### 3.3. The Protection of Spirulina on the Visual Function 

Light-induced retinal damage is characterized by the accumulation of ROS, leading to oxidative stress and photoreceptor cell death. The light rays acting on the retina stimulate a vastness of receptors to transform the light message into electrical impulses. On the other hand, ROS are also generated. The latter may induce oxidative damage to retinal photoreceptors. In mice, it has been seen that therapy with Spirulina can reduce the production of ROS and protect visual functions from wasting [89]. Spirulina has the potential as a nutrient supplement to prevent vision loss related to oxidative damage in the future [89]. The use of natural antioxidants has emerged as a promising approach for preventing light-induced retinal damage. However, less is known about the possible efficacy of combining natural antioxidants in a multicomponent mixture. Lutein and cyanidin-3-glucoside (C3G) are particularly effective due to their antioxidant and anti-inflammatory activity. These findings suggest the rationale to formulate multicomponent blends, which may optimize the partnering compounds’ bioactivity and bioavailability [90]. Age-related macular degeneration (AMD) is a significant visual impairment in older people, and there is no treatment for dry AMD. In-vitro, Spirulina decreased blue light-induced retinal pigment epithelium (RPE) cell death by inhibiting ROS production and inhibited BL-induced inflammation via regulating the NF-κB pathway, inflammatory-related gene expression, and the apoptosis pathway in RPE cells. In vivo, the administration of Spirulina inhibited blue light-induced retinal degeneration by restoring the thicknesses of the whole retina, ONL (outer nuclear layer), INL (inner nuclear layer), and PL (photoreceptor layer) by BL exposure. Therefore, Spirulina could be a potential nutraceutical approach to intercept the pathophysiological processes leading to dry AMD and advancement to wet AMD [91].

## 4. Applications in Oncology

### 4.1. Breast Cancer

High delivery efficiency, prolonged drug release, and low systemic toxicity are effective weapons for drug delivery systems to win the battle against metastatic breast cancer. It is demonstrated that Spirulina can be used as a natural carrier to build a drug-loaded system for targeted delivery and fluorescence imaging-guided chemotherapy on breast cancer lung metastases [92]. The *Spirulina’s* protein C-phycocyanin (C-PC) inhibited cell proliferation and reduced the colony formation ability of MDA-MB-231 cells. Furthermore, C-PC induced cell cycle G0/G1 arrest by decreasing protein expression levels of Cyclin D1 and CDK-2 and increasing protein expression levels of p21 and p27. In addition, C-PC induced cell apoptotic by activating the cell membrane surface death receptor pathway. Besides, C-PC down-regulated the protein expression levels of cyclooxygenase-2 and further inhibited MDA-MB-231 cells migration [93]. Additionally, a study tested two nutritional supplements, namely gamma-tocotrienol (γT3) and Spirulina, for their immune-enhancing and anticancer effects in a syngeneic mouse model of breast cancer. It has been assessed that combined γT3 + Spirulina treatment did not show any synergistic anticancer effects in this study model [94]. Also, CD4, CD8, and CD56 staining were performed to investigate the effect on the immune cells’ recruitment to the tumors by immunohistochemistry. This treatment combination could significantly inhibit 4T1 breast tumors’ growth, decreasing the tumors’ volume compared to the control and the metastatic burden [92].

### 4.2. Hepatocarcinoma

Most cases of hepatocellular carcinoma (HCC) are diagnosed in the advanced stages of the disease, making it the second leading cause of cancer mortality worldwide. For advanced patients, chemotherapy drugs are the best treatment option; however, their adverse effects and high cost are still the main obstacles to effective treatment. Spirulina is a rich source of nutritional and bioactive elements and potential pharmaceuticals and has an anti-proliferative effect against several tumor cell lines. It also has a prophylactic effect against the early stages of certain cancer models, including HCC. Spirulina inhibited structural and functional alterations of HCC, manifested by improving the survival rate, significantly decreasing the tumor marker AFP and the count and size of liver nodules, as well as reducing HCC [95]. This was accompanied by increased endogenous antioxidant capacity, apoptosis (Bax), and tumor suppressor protein (p53), as well as suppression of tissue levels of lipid peroxidation marker (MDA) and neoangiogenesis marker (VEGF). Spirulina has an anti-carcinogenic effect against advanced HCC exerted by activating the tumor suppressor protein p53 and apoptosis and suppressing oxidative stress and angiogenesis [95]. The L-AsnA enzyme, contained in Spirulina, in a recent study, showed an antiproliferation effect against lung cancer A549, hepatocellular carcinoma Hep-G2, and prostate cancer PC3 human cancer cell lines. Considering the antiviral and antiproliferative activity of L-AsnA against different human cell lines, it would be desirable to investigate its effects further [81].

### 4.3. Lung Cancer

The anticancer potential of a hot water extract of a commercial Spirulina against the human non-small-cell lung carcinoma A549 cell line has been evaluated in the literature [96]. Spirulina significantly reduced cancer cell viability and proliferation, which was accompanied by cell cycle inhibition in the G_1_ phase, induction of apoptosis, and prominent morphological changes. Moreover, it has been detected that there is no cytotoxic effect of the tested Spirulina on normal skin fibroblasts. The evidence is the anticancer activity of the Spirulina against lung cancer cells and strongly supports the knowledge of the chemo-preventive properties of Spirulina [96].

## 5. Applications in Central and Peripheral Nervous System

It has been shown by several scientific studies that cyanobacteria, especially Spirulina, are rich in micro and macronutrients important for brain health, such as B vitamins, particularly vitamin B12, amino acids, and minerals such as iron, calcium, zinc, magnesium, manganese, and potassium [97,98,99,100,101]. The B vitamins can reach the brain, and in fact, like the other bioactive derivatives of cyanobacteria, they control various neuronal functions both through epigenetic mechanisms that control gene transcription neurotransmitters and through their antioxidant and anti-inflammatory abilities [102]. The beneficial effects of Spirulina on the Central Nervous System (CNS) are also derived from the interaction between the Spirulina phytocomplex and the intestinal microbiota; there is a close relationship between the nervous system and the bowel, the bowel–brain axis [103]. The microbiota can transform Spirulina into antioxidant and micronutrient molecules that are able to cross the blood–brain barrier and express their beneficial effects such as the increase in energy production and the reduction of mental fatigue with the enhancement of learning skills; the synthesis of neurotransmitters (dopamine, serotonin, glycine, glutamate/GABA); an overall improvement in cognitive functions and short- and long-term memory [104]. On the other hand, Spirulina can increase the growth of protective bacteria in the bowel, thus maintaining a good balance and intestinal integrity and reducing systemic inflammatory responses that have a negative impact on the nervous system [105,106]. Alterations of the microbiota have been demonstrated in many neurological disorders, and the use of probiotics and prebiotics such as those in the Spirulina phytocomplex can indirectly prevent the development of brain disorders [107].

In neurodegenerative diseases such as Parkinson’s or Alzheimer’s and other psychocognitive diseases, Spirulina plays a neuroprotective role thanks to the antioxidant capacity of its derivatives, as demonstrated by numerous in vivo studies on animals [108,109,110,111,112]. In Alzheimer’s disease, Spirulina can prevent memory loss by reducing the deposition of β-amyloid in the brain and increasing the activity of glutathione peroxidase and catalase. The immunomodulatory and antioxidant effect of Spirulina has also been demonstrated in humans. Park et al. observed how cholesterol levels were lower in elderly patients who took Spirulina daily, while those of superoxide dismutase, IL-2, and IL-6 increased [113]. During adolescence, Spirulina can reduce stress-related disorders and the induced remodeling of limbic structures, in particular the amygdala, with the consequent lower risk of neuropsychiatric disorders in adulthood [112,113].

Further human studies have highlighted an improvement effect in cognitive abilities and neuronal activation, with an increase in memory and attention [114,115]. Finally, Spirulina is an important nutritional source in cases of severe malnutrition, especially protein malnutrition (PNM), a frequent phenomenon very common in the third world countries which causes detrimental effects on the brain development of malnourished children particularly in the hippocampus. Penton-Rol et al. [116] demonstrated that C-PC, a biliprotein derived from Spirulina with anti-inflammatory, antioxidant, and cytoprotective capacities, had neuroprotective effects on mice affected by experimental autoimmune encephalomyelitis. Such effects are due to the ability of C-PC to reduce the activation and infiltration of lymphocytes and macrophages/microglia activated in case of autoimmune encephalomyelitis. These cells are also responsible for the pathogenesis of multiple sclerosis, as they result in the production of neurotoxic molecules, proinflammatory cytokines, and present self-antigens that cause demyelination [117,118]. Penton-Rol et al. [116] demonstrated, in the animal model, that C-PC can promote significant axonal remyelination, as well as reduce the Amyloid Precursor Protein (APP), a marker protein of Alzheimer’s disease. This is an encouraging result for the treatment of this neurodegenerative pathology. The data described are also encouraging for possible future clinical applications of Spirulina in the field of nerve regeneration and/or reconstructive nerve surgery [119,120,121]. 

## 6. Applications in COVID-19 Infection

The outbreak of the 2019 coronavirus disease (COVID-19), caused by the severe acute respiratory syndrome coronavirus 2 that has created enormous trepidation worldwide, has a mortality rate of 0.5% to 1% and is growing incessantly. Prior to the commercialization of the COVID-19 vaccine, several areas were launched in order to find better treatments for this infection. In this context, the ability of cyanobacteria-based nutraceuticals, mainly Spirulina, to increase immunity against viral diseases was studied; already widely reported clinically. Spirulina-based nutraceuticals increase adaptive and innate immunity, and bioactive compounds, such as ACE inhibitor peptides, phycobiliproteins, sulfate polysaccharides, and calcium spirulan, can serve as antiviral agents. The presence of these molecules indicates its potential role in resistance to infection and progression of the COVID-19 disease. Such cyanobacteria-based nutraceuticals could be used as immune boosters to fight the human coronavirus and other viral diseases in association with vaccines [122]. The spike protein of severe acute respiratory syndrome due to COVID-19 uses angiotensin-converting enzyme 2 as the receptor for cell entry, which is highly expressed in the gut and lungs. Nausea and diarrhea are the primary symptoms of COVID-19 even before the development of fever and respiratory symptoms. These two tissues share a relationship influencing inflammatory and immune responses via the gut–lung axis that can be responsive to probiotics through effects on commensal microbial flora. Some probiotics enhance regulatory T-cell activity and reduce pro-inflammatory cytokine production. Some probiotics have shown antiviral protective and therapeutic effects regarding upper respiratory tract disease, lessening the severity and extent of tissue damage from infection and inflammation. Evidence suggests that probiotics with anti-inflammatory or immunomodulatory properties might be predicted to have the most beneficial potential to prevent or alleviate COVID-19 symptoms [123].

## 7. Interaction with Stem Cells

Several studies have shown that nutraceuticals exert effects on adult stem cells. At the same time, robust evidence supports the therapeutic benefits of Spirulina in clinical settings due to its antioxidant and anti-inflammatory properties [124,125]. In the last decade, in vitro and in vivo studies have reported other effects, such as antihyperlipidemic [126], anticancer [127], anti-neurotoxic [128], and anti-type 1 diabetic [129] properties. Regarding the immune system, a review of the main immunomodulatory and anti-inflammatory properties, C-PC was one of the most abundant phycobiliproteins of Spirulina. The C-PC has been used in biomedical research as a biomarker for its fluorescence properties. It has been shown that this protein increases the release of γ-interferon in peripheral blood mononuclear cells and modulates the production of inflammatory cytokines such as tumor necrosis factor, among others. Furthermore, C-PC has immunomodulatory effects on cytokines that enhance the activation of immune cells, such as IL-6 and IL-1β, and the regulation of about 190 genes involved in immunity [80]. 

In addition, using Spirulina biomass for in vitro cell cultures has become feasible in biotechnological research due to its well-established nutrient-rich properties, sustainability, and ethically acceptable source. It has been considered a novel alternate supplement for fetal bovine serum (FBS). Recently, *Spirulina* animal cell culture solution showed a better growth-promoting capability than FBS, demonstrated as an effective, low-cost, and eco-friendly substitute to FBS in vitro cultures of H460 cells [130]. In preclinical studies, it was also demonstrated the chemo- and radio-protective effects of Spirulina polysaccharide on the hemopoietic system of mice and dogs [130], and the regression of tumors in animal models [124], while other papers pointed out that Spirulina was able to reduce the deleterious effects of acute inflammatory insult on neural progenitor cells functions [131]. Furthermore, other authors have demonstrated that stem cells cultivated in scaffolds associated with Spirulina biomass adhered more and had greater viability when compared with the scaffold alone [131]. Perhaps most interesting regarding the clinical use is the ability to assist with stem cells mobilization. In 2006, the effects of nutraceuticals compounds on stem cells as a viable alternative to induce the proliferation and mobilization of human bone marrow and human CD34+ and CD133+ cells were explored [132]. Another study evaluated human stem cells in vitro and in vivo of an extract from the edible cyanobacterium *Aphanizomenon flos-aqua* (AFA) [133]. This paper includes a double-blind, randomized crossover study involving 12 healthy subjects that evaluated the effects of consumption on stem cell mobilization and found that it produced a better ability for stem cells to travel to the tissues where they are most needed. The study reported that oral consumption of a supplement comprising a blend of two compounds extracted from AFA triggered an average 25% increase in the number of peripheral blood stem cells within 60 min after consumption. The magnitude of this mobilization on CD34+ cells was smaller than that triggered by Granulocyte-Colony Stimulating Factors with marketing authorization, but its safety allows for continuous use. Therefore, considering the numerous advantages of this novel approach in stem cells mobilization, the Abu Dhabi Stem Cells Center Research Team is developing basic research studies and clinical trials for further clinical application in the mobilization of hematopoietic stem cells to peripheral blood [125,134].

## 8. Nephrological Involvement

Renal toxicity is a restricting factor that affects the use and the dosing of many drugs and agents with disparate use in human medicine as antibiotics, chemotherapeutics, radiological contrast media, and immunosuppressive agents. Oxidative stress plays a key role in many of them. Spirulina and its antioxidant effects have been studied against several agents with known nephrotoxic potential; here, we briefly report the main results available in the current literature. Renal toxicity of oncological therapy is a major field of research, as it often brings about a reduction in drug dosage and the subsequent under-treatment of patients with renal impairment. A similar statement can be applied to antibiotics such as aminoglycosides, especially in countries where the availability of the most recent drugs is difficult and cost-biased. Spirulina seems to have the potential to significantly improve renal outcomes in these patients. In 2006, Kuhad et al. [134] evaluated renoprotective effects against Cisplatin-induced oxidative stress in the murine model. Six groups of rats were treated with a placebo, cisplatin alone, Spirulina alone, and cisplatin associated with an increasing dose of Spirulina (500, 1000, and 1500 mg/kg, from two days before and continually until three days after cisplatin administration), respectively; both biochemical and histological parameters were evaluated. Results showed a Spirulina dose-dependent attenuation of oxidative stress via a reduction of lipid peroxidation (LPO), leading to improvement in serum creatinine and urea clearance values; histology documented a reduction in the severity of morphological kidney damage. Avdagiće et al. [135] studied the efficacy of Spirulina in the reduction of gentamicin-induced nephrotoxicity in rats, demonstrating a significant reduction in plasma nitric oxide (NO) levels in rats treated with Spirulina and lighter signs of acute tubular necrosis on histology. Similar results have been reached by Hamad et al. [136], confirming beneficial effects on kidney histology and oxidative stress markers (MDA, GSH). Calcineurin inhibitors (CNI) are a class of immunosuppressant drugs currently used in several auto-immune disorders and to avoid rejection in solid organ transplantation. The most frequently used CNI’s are cyclosporin A (CsA) and tacrolimus, both suffering from a known and dose-dependent nephrotoxicity; several mechanisms are involved in CNI renal damage, including oxidative stress and nitric oxide-related hemodynamic alterations caused by the activity of the inducible form of nitric oxide synthase (iNOS) in renal tissues. Khan et al. [137] found that, in rat models, the antioxidative effects of Spirulina prevented the rise in plasma creatinine and urea values as well as the severe isometric vacuolization and widening of the interstitium on histology. Furthermore, Spirulina does not interfere with the CsA metabolism. Moreover, Spirulina seems to have vasodilating properties on rat aortic rings, probably related to the cyclooxygenase-dependent product of arachidonic acid and nitric oxide (NO) [138], which could lead to improved renal perfusion; nevertheless, specific studies on effects in renal vasculature and blood flow has not been reported yet. Regarding renal drug-induced toxicity, oxidative stress plays a pivotal role in the progression of chronic kidney disease, one of the leading causes of morbidity worldwide as well as mortality and high medical costs among adults. Memije-Lazaro et al. [139] have assessed the beneficial effects of Spirulina in chronic kidney disease (CKD) rat models, obtaining significant reductions in oxidative stress and hypertension, leading to protection against cardiovascular and renal alterations. This is a brief overview of several potential benefits of Spirulina in different scenarios of kidney damage. All the studies reported were conducted in vivo in animal models (e.g., rats) and require more validation to confirm results on human tissue (both in vivo and in vitro) [81,133,139]. 

## 9. Beyond Earth: Space Sustainability

**Figure 2:**
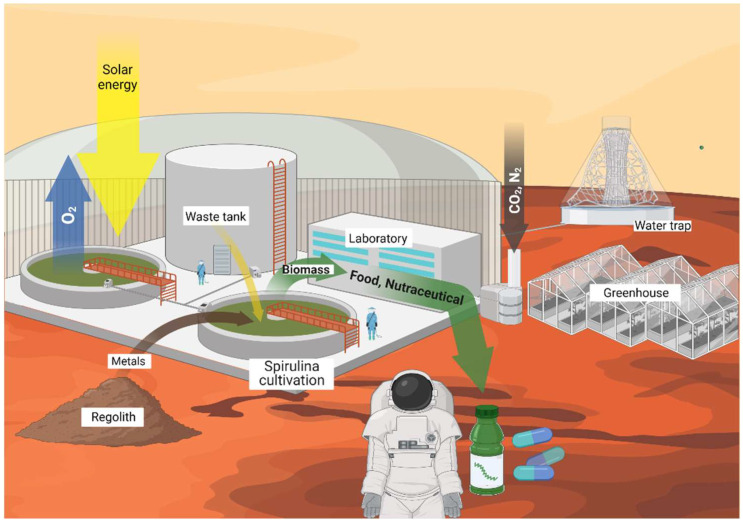
Rendering of ECLSS and ISRU systems for the cultivation and utilization of *Spirulina* on Mars. Created with BioRender.com (accessed on 8 March 2022).

Earth resources depletion, overpopulation, climate crisis, and pandemics call for the identification of new strategies to save the future of humanity, which involve the use of renewable energies and materials, recycling of wastes, water savings, economic degrowth, etc. While these strategies can help in the short-medium term, it is well established that, in the long term, humanity should be capable of travelling and living on other planets to survive [140]. Among the planets where human life might realistically be possible in the midterm, Mars represents the strongest candidate due to its proximity to Earth. In addition, the temperatures are close to those of continental winters (~−14 °C on average on the equator), and the day duration (~25 h), average solar irradiance levels (~20 mol m^−2^ sol^−1^), and presence of resources such as atmospheric CO_2_, water, and regolith, which might be transformed and exploited in-situ to produce useful consumables [141] (Figure 2). 

For these reasons, the main space agencies, gathered in the International Space Exploration Coordination Group (ISECG), have listed manned missions to Mars as a common target [60].

The accomplishment of this goal necessitates the identification of new technologies for the sustainment of long-duration manned missions to planets beyond the Lower Earth Orbit (LEO) [142]. So far, several studies have been devoted to developing Environmental Control and Life Support Systems (ECLSSs), typically involving microalgae and cyanobacteria, which permit the production of water, oxygen, and food by totally recycling crew metabolic wastes, including exhausted cabin air [56,57,58,143]. In this regard, *Spirulina* is recognized as a crucial constituent of the MELiSSA project due to its high photosynthetic efficiency, reaching values close to 6% and protein content of 60–70% wt [142]. Moreover, *Spirulina* can use nitrogen available in the urine water through urease-catalyzed reactions that convert urea into NH_4_^+^ and bicarbonate, making it particularly useful for recycling astronaut’s urine [144]. Moreover, it has been estimated that about ~93% of O_2_ and 50–100% of the food needed by astronauts could be produced in the ECLSSs [145]. For this reason, this species is considered crucial in the framework of the new generation ECLSSs by the European Space Agency and has been successfully grown in the International Space Station (ISS) in 2017.

On the other hand, given its use on other planets, the current ECLSSs are not completely self-sustaining and thus require the integration of external inputs of supplies to produce the amount needed to meet the astronauts’ needs [54,58,141]. In fact, since interplanetary trips are quite expensive [60], the integrative amounts of consumables cannot be continuously replenished from Earth but must be produced directly on Mars by exploiting the approach represented by the acronym ISRU (In Situ Resource Utilization). Therefore, the ideal strategy for sustaining long-duration manned missions beyond LEO involves the synergistic coupling of ECLSSs and ISRU technologies [56].

While the use of bio-engineering techniques involving cyanobacteria, microalgae, macroalgae, bacteria, and fungi is currently well established in the realization of bioregenerative ECLSS [56,60,142,143], very few works exist in the literature dealing with the possibility of using it to transform available in-situ resources such as regolith and atmospheric CO_2_ into useful supplies on Mars. In fact, most ISRU technologies so far proposed consist of physic-chemical methods for oxygen and propellants production from Martian regolith and atmosphere but cannot contribute to the production of food [146,147]. On the other hand, in-situ food production is the main bottleneck for the manned mission since ECLSSs can contribute only to a limited extent to the needs of a crew, estimated to be around 3000 cal sol^−1^ when the latter one is composed of six members [141]. Thus, ISRU technologies for food production on Mars are needed. Albeit several works envision the cultivation of crops, cyanobacteria, algae, and fungi on Mars, only a few papers addressed the transformation of Martian resources into edible biomass under operating conditions that simulate the Martian ones.

In this regard, the few bio-ISRU technologies far proposed relies on rock weathering cyanobacteria which can photosynthetically convert N_2_ and CO_2_, along with S, P, Fe, and several micronutrients, available in the Mars atmosphere and the regolith, respectively, into newly formed edible biomass by relying on the water and the light available in-situ [60,148]. The use of cyanobacteria and microalgae leads to the further positive effect of producing photosynthetic oxygen, which is crucial for the crew and can integrate the amounts of oxygen produced via physic-chemical methods.

The possibility of using several cyanobacteria exposed to simulated Martian conditions (−27 °C, 0.8 kPa, pure CO_2_) has been investigated by Olsson-Francis and Cockell [148]. The experimental results indicated that the strains *Anabaena cylindrica*, *Chroococcidiopsis 029*, *Gloeocapsa OU_20*, *Phormidium OU_10,* and *Leptolyngbya OU_13* were able to survive several days under Mars simulated conditions and using a regolith simulant as growth substrate [148]. Therefore, albeit neglecting the effects of microgravity, this study demonstrated that cyanobacteria could be used for ISRU purposes. 

Recently Verseux et al. [60] investigated the diazotrophic growth of *Anabaena* sp. PC7983 under an artificial low-pressure (~101 hPa) atmosphere composed of N_2_ (96%) and CO_2_ (4%), which the authors envisioned to be produced by using the Martian atmosphere. The results showed that this strain could vigorously grow by taking C and N from such an atmosphere and other micronutrients from a Martian regolith simulant immersed in the growth medium cyanobacteria (BG-11) [60]. Growth experiments under low-pressure atmospheres (80–670 mbar) consisting of pure CO_2_ to simulate Martian one were performed by Cycil et al. [144], but they found a scarce capability to survive under such low pressure due to the onset of the carbon starvation phenomena.

In the work by Billi et al. [149], the capability of the autotrophic strain *Chroococcidiopsis* sp. to tolerate perchlorate salts, typically found in Mars regolith, was investigated. The obtained experimental results showed that exposition to Mars-relevant concentrations of Mg or Ca perchlorate did not affect the growth of this strain under simulated Martian conditions, thus demonstrating that *Chroococcidiopsis* is a good candidate for bio-ISRU contexts on Mars.

To the best of our knowledge, aside from the works cited above, only a few less comprehensive papers dealing with the use of Mars environmental resources to sustain the growth of microalgae and cyanobacteria can be found in the literature. Moreover, all these papers had the common lack that the effect of microgravity (~1/3 g on Mars) was neglected during the experiments. Thus, further research activity is needed to verify the possibility of using microalgae and cyanobacteria as a potential food source in the framework of manned missions on Mars that rely on ISRU technologies.

Recently, a novel process has been proposed to grow *Spirulina* on Mars while exploiting available in-situ resources such as atmospheric CO_2_ and regolith. The process, which should take place within pressurized and heated domes, also takes advantage of the urine produced by crew members to produce the growth medium wherein it is cultivated. The performances of the proposed process have been evaluated on Earth through an apparatus patented by Cao et al. [150]. It is shown that microgravity was beneficial for *Spirulina* growth because it inhibited the aggregation of cells, thus favoring the transfer of nutrients from the liquid bulk to the cells. At the same time, the high CO_2_ content in the gas phase, which simulated the composition of the Martian atmosphere, was able to prevent carbon starvation phenomena, thus prolonging the growth with respect to the case where air CO_2_ concentration (0.038% vol) was used. Ultimately, using the process invented by Cao et al. [150], *Spirulina* seemed to grow better under simulated microgravity conditions than on Earth. From the obtained results, it was extrapolated that a culture of 6 m^3^ could be enough to meet the 40% of the protein needs of a crew consisting of six members. These encouraging results open the way to using Spirulina as a food source to be produced in situ during the future manned missions on Mars.

## 10. Spirulina for Astronauts’ Healthcare 

During their stay in space, astronauts are exposed to a variety of stress factors that can have negative health consequences even after years. With the acronym “RIDGE” (space radiation, isolation and confinement, distance from Earth, gravity fields, and hostile and closed environments), NASA indicates the main dangers of space flights. For instance, with the advent of space stations, which are habitable for long periods, it has been widely demonstrated that exposure to microgravity has several negative effects on human health. These effects may even have a long-term impact, affecting gastrointestinal diseases, including relevant effects on the composition of astronauts’ microbiota [151,152,153,154,155,156,157,158,159,160], thermoregulation, heart rate, muscle tone, respiratory system, and other physiological aspects of the human body [51]. Some of the key health risks to astronauts in a microgravity environment include musculoskeletal changes such as reduced muscle strength and increased bone fragility, visual impairment, endothelial dysfunction, metabolic changes, behavioral changes due to fatigue or stress, and effects on mental well-being.

Astronauts in space are exposed to high levels of cosmic radiation, which can damage the DNA, mutations, oxidative stress, acute radiation syndromes, central nervous system injury, tissue diseases such as cardiovascular disease, and the composition of the gut microbiota. More specifically, one of the main effects of space flight at the molecular and intracellular level is the onset of oxidative stress, which causes DNA and mitochondrial damage [161,162]. Oxidative stress leads to an imbalance between the production and disposal of highly pro-oxidant radical species such as ROS and nitrogen, which have several cellular targets: cytoplasmic, mitochondrial, and nuclear, including nucleic [163,164]. Closely related to oxidative stress in mitochondrial dysregulation, identified by the reduction in the expression of genes involved in oxidative phosphorylation of the mitochondria. The latter one is, in fact, the main endogenous production site of ROS, hence its increased vulnerability to the onset of oxidative stress [162,165]. In space, there is a high production of oxidant species due to the extreme conditions to which crew members are exposed, leading to an imbalance between oxidant and antioxidant species. The most produced ROS are superoxide anion (O^2−^), hydrogen peroxide (H_2_O_2_), and free radicals. An increase in ROS leads, as in radiation damage, to DNA mutations. ROS and cosmic radiation are among the major causes of ageing in space because of the damage they induce in the body. It has been demonstrated in the literature that the use of a Spirulina supplement can increase antioxidant capacity and thus counteract ROS production. 

Space environments expose astronauts to a high level of oxidative stress due to extravehicular and intravehicular activities (EVA/IVA) and depend on reduced gravity, ionizing radiation, and variable atmospheric conditions, such as hyper- and hypoxia and psychophysical stress [166,167,168,169]. In addition, several studies show how astronauts experience immunodeficiency with impairment of NK cells, redox imbalance, elevated inflammation, elevated granulocytes, inhibited lymphocyte proliferation, and reduced lymphocyte functions, as well as elevated inflammation during low-Earth orbit missions [170,171,172,173,174]. 

During space missions, oxidative stress and mitochondrial dysfunction caused by exposure to cosmic rays are responsible for renal insults at multiple levels and have negative consequences on the physiology of the kidney [175]. In addition, changes in fluid balance and electrolytes occur due to microgravity conditions, which alter hemodynamics and the organ function [172,176,177]. Electrolyte imbalances increase the chance of developing kidney stones in astronauts [178,179]. Histological analysis of rats exposed to microgravity showed glomerular atrophy, interstitial edema, and degeneration of renal tubules [180].

In terms of the physiology of the human body, space flights induce effects on the cardiovascular, immune, and nervous systems, which also increases the risk of cancer. Astronauts are partially protected against cosmic radiation and from rare solar events, agents which can cause biological changes at the molecular level. [51,181,182]. At the physiological level, space flight primarily produces a displacement of biological fluids to the upper part of the body. This displacement determines an increase in the excretion and activation of compensation mechanisms by the cardiovascular system, which leads to the so-called “deconditioning of the cardiovascular system”, characterized by reduced blood volume, variation in cardiac contractility, stiffening of arterial vessels, and the development of insulin resistance [183]. Microgravity reduces the heart’s ability to pump blood and provide oxygen to tissues. The heart adapts to changes in blood distribution and pressure in microgravity, and the response of heart rate adaptation to changes in blood pressure is different among astronauts. In addition, arterial stiffening in space causes an increase in blood pressure that can lead to an increased risk of cardiovascular disease. The space environment can also cause the acceleration of vascular aging [184].

The extraterrestrial stressors also cause neurobiological imbalances at several levels due to molecular, biochemical, and cellular perturbations that involve the architecture of neural circuits, the expression of proteins, and synaptic plasticity, also determining neuroinflammation [185,186]. Radiation can cause deficits in cognition, learning, memory, attention, and executive functions, resulting in mood changes such as anxiety and depression [187,188,189,190]. Recent studies have linked these disorders to neuronal structural changes, variations in the microcirculation, and neuroinflammation, and it has been shown that radiation leads to an increased presence of dense fibrillar proteins and β-amyloid [191,192,193]. Radiation exposure reduces the glutamatergic vesicular pool in synaptosomes and reduces the expression of the N-methyle-d-aspartate (NMDA) glutamatergic receptor subunits [194], with possible repercussions on the excitatory/inhibitory state of the nervous system. Regarding the effect of microgravity on the physiology of astronauts, it has been observed that microgravity determines a variation in the architecture of the neuronal cytoskeleton, resulting in imbalances at the level of biochemical and biosynthetic pathways and altering DNA replication, RNA transcription, and protein transport [195,196,197,198].

Additionally, neurological deficits in space are closely linked to the immune dysregulation induced by space flight, which leads to a strong exacerbation of inflammatory phenomena [199]. Immune dysregulation consists of a change in the function of certain cells in the immune system (T-cells and natural killer cells) and in the expression of cytokines [159,200,201]. The resulting persistent moderate inflammation is the basis for the reactivation of latent viruses in astronauts’ bodies, which increases the risk of cancer [202]. During space flights, astronauts are exposed to an increased risk of cancer due to their increased exposure to hazardous factors such as ionizing radiation, solar particle events, and the Van Allen belt. Among the various damages caused by radiation, the one caused to DNA is quite important, and although the body has repairing mechanisms, they can still create irreversible conditions leading to, among other things, the development of tumors [203,204,205,206].

Among the varieties of proteins that Spirulina contains, the study of C-PC proteins brought clinically relevant information. One of the main characteristics of C-PC is the fluorescence capacity which makes Spirulina very adaptable for diagnostic purposes, specifically in the case of tumors, e. g. breast and lung cancer. This protein also promotes the release of γ-interferon in peripheral blood mononuclear cells, thus directly stimulating the immune system. In the same way, it can stimulate the immune system and modulate the production of inflammatory cytokines such as tumor necrosis factor. The extract of Spirulina showed antioxidant activity depending on the concentration. This extract can have interesting effects on the cell cycle of lung cancer. Results showed that it could stop the growth of cancerous cells in phase G by preventing the passage in phase M, thus blocking, in fact, tumor growth [207].

Another problem encountered by astronauts during space flights is a decrease in circulating red blood cells (RBCs), leading to anemia, which is thought to be due to an inhibition of bone marrow function resulting in the decrease of erythropoietin production. It should be noted that the decrease in circulating RBCs is an acute adaptation to the hemodynamic events of cephalic fluid shift due to weightlessness [52,208]. Anemia leads to a reduced ability of red blood cells to transport oxygen and a reduction in the amount of hemoglobin, which is reflected in an impaired ability of the body to exchange gases. Studies have shown that Spirulina, due to its Vitamin B12 and iron content, can somehow counteract anemia [209]. 

Also, ophthalmic health in astronauts is compromised during long-term space flights, and clinical manifestations include an increased risk of cataracts, optic disc edema, globe flattening, hyperopic shifts, choroidal folds, and cotton wool spots [210]. Causes are not known but some studies suggest that vitamin B_9_ (folate) and vitamin B_12_ may be involved in these dysfunctions, and proper nutrient intake may improve them [211,212]. Folate deficiency could have negative consequences during space missions. In fact, it could cause chromosomal damage due to the increased sensitivity to ionizing radiation present in space [213]. In addition, folate has been shown to eliminate a wide range of ROS efficiently [214,215]. Some research supports the idea that changes in the metabolic pathway of vitamin B_12_ could lead to ophthalmic problems such as optic neuropathy and age-related macular degeneration [212].

Space environments can influence the development of acquired immunity and immune responses. In fact, during space missions, most astronauts experience deficiencies in their immune system. Since the development of coronavirus disease 2019 (COVID-19), which is more severe for those immunocompromised, NASA has recently implemented clinical testing and monitoring to protect international space station astronauts [216,217].

Moreover, during space missions, experiments have been carried out on stem cells that demonstrate a change in their ability to differentiate when exposed to microgravity. These experiments performed on the space shuttle have shown that microgravity impairs the differentiation of stem cells [218,219].

*Spirulina* has already been included in the study programs of space agencies such as NASA [220,221]. Many of the problems faced by astronauts during their missions are exacerbated by the poor quality of food, which is crucial to stay healthy and protect themselves against the effects of microgravity. It is difficult and very expensive to continuously provide fresh food to crew members from Earth. Since on the ISS, it is not possible to keep fresh at room temperature, it must be rehydrated or cooked to be consumed. Lack of fresh food, such as fresh fruit and vegetables, leads to a low intake of macro- and micro-nutrients essential for health, and the limited amount of food available does not allow for a proper daily caloric intake. In recent years, space agencies and other companies have been investigating the possibility of producing food using the so-called ISRU, which involves microalgae to produce food and water in space to have available fresh food. Among the microalgae species currently known in the literature to be suitable for human intake is *Chlorella vulgaris* which have been declared safe for human consumption by the Food and Drug Administration (FDA) and has been placed in the GRAS category [222]. Spirulina is currently widely used not only in space but also on Earth due to its high protein content with a complete amino acid profile and high content of macro- and micro-nutrients that make it excellent for use as a food source [142,222].

## 11. Conclusions

Overpopulation and the depletion of the Earth resources is leading to limited consumption of natural food in the human population, thus causing a shortage of macro- and micronutrients, including vitamins and antioxidants. Along with the increase in population, the demand for quality food and the usage of food supplements has increased. People have become more aware of the link between proper nutrition and personal health. There are also many different diseases that humans can suffer, both on Earth and in space. These diseases can be caused by genetic alterations, endogenous and exogenous factors such as ionizing and cosmic radiation, changes in the composition of the microbiome, and the effects of microgravity in space environments. Specifically, proper nutrition has proven to be a valuable aid in the prevention and treatment of the most important diseases of the world. Cyanobacteria such as *Spirulina* play a key role in this contest since it is well known that its sustainable cultivation for the food and nutraceutical industry not only benefits Planet Earth but is also a potential solution to counteract malnutrition. NASA was the first space agency to conduct experiments in space using *Spirulina* as a food source for astronauts. To date, its documented effects are manifold, its high protein content, characterized by a complete amino acid profile including all the essential amino acids, and its excellent supply of vitamins such as the B vitamin complex, Vitamin C and E give it antioxidant properties. Numerous studies conducted on Spirulina have shown that its use can improve health due to its high-value composition of micro and macro nutrients. In addition, its use as a supplement to improve the health of the intestinal microbiota has anti-cancer and anti-inflammatory effects and can help strengthen the immune system. For all these positive effects on the organism, *Spirulina* has been included in the ECLSSs and ISRU technologies of the major space agencies. This brief review also shows that the intake of Spirulina by astronauts may play a crucial beneficial role in enabling a longer and safer stay of humans in space.

## Figures and Tables

**Table 1:** *Spirulina platensis* main compounds.

**Macronutrients**	**% d.w.**
Proteins	50–70
Carbohydrates	15–20
Lipids	7–16
**Vitamins**	**mg/100 g d.w.**
Carotene	140
Biotin	0.005
Folic acid	0.01
Niacin	14
Riboflavin	4
Thiamin B1	3.5
Vitamin E	100
Vitamin B12	0.32
Vitamin K	2.2
**Carbohydrates**	**mg/100 g d.w.**
Glucose	54.4
Galactose	2.6
Mannose	9.3
Rhamnose	22.3
Xylose	7
**Lipids**	**g/100 g d.w.**
Arachidic	0.048
Gamma linolenic (GLA)	1
Myristic	0.041
Oleic	0.017
Palmitic	2
**Amino acids**	**g/100 g d.w.**
Leucine	4.94
Isoleucine	3.2
Lysine	3.02
Methionine	1.15
Phenylalaline	2.78
Threonine	2.97
Tryptophan	0.93
Valine	3.51
**Phytonutrients**	**g/100 g d.w.**
*cis* beta-carotene	0.073
Chlorophyll-a	1
Phycocyanin	12
*trans* beta-carotene	0.26
**Minerals**	**mg/100 g d.w.**
Calcium	700
Copper	1.2
Iron	100
Magnesium	400
Manganese	5
Phosphorus	800
Potassium	1400
Sodium	900
Zinc	3

## Data Availability

Not applicable.
